# Beneficial Effects of Vitamin C in Maintaining Optimal Oral Health

**DOI:** 10.3389/fnut.2021.805809

**Published:** 2022-01-10

**Authors:** Julienne Murererehe, Anne Marie Uwitonze, Pacifique Nikuze, Jay Patel, Mohammed S. Razzaque

**Affiliations:** ^1^Department of Preventive and Community Dentistry, School of Dentistry, University of Rwanda College of Medicine & Health Sciences, Kigali, Rwanda; ^2^Department of Pathology, Lake Erie College of Osteopathic Medicine, Erie, PA, United States

**Keywords:** oral - general health, vitamin C (ascorbic acid), dental decay, gingivitis, oral tumor

Vitamin C, also known as ascorbic acid, is typically obtained from the diet. Small amounts of water-soluble vitamin C are needed to maintain normal body functions (1–3). Vitamin C has been broadly recognized as the most important hydrophilic antioxidant and is a specific cofactor for many enzymatic reactions. Most plants and animals can synthesize vitamin C from D-glucose and D-galactose. However, due to the absence of the enzyme L-gulonolactone oxidase (GLO) in humans and some animal species such as monkeys, guinea pigs, bats, and birds, they are unable to generate endogenous forms of vitamin C (2, 4). For that reason, humans must obtain vitamin C from their diet or take supplements because a total deficiency of vitamin C in humans can result in spongy swollen bleeding gums, dry skin, open sores on the skin, fatigue, impaired wound healing, and depression. Scurvy can occur when healthy individuals consume <10 mg of vitamin C per day. Additionally, some cancers, anemias, and infections have been linked to a vitamin C deficiency (3, 5, 6). Ascorbic acid is sensitive to air, light, and heat, thus can be destroyed by overcooking and storing food for prolonged periods. In addition, vitamin C is not stored in the body, which is the reason why it must be regularly consumed.

The highest levels of vitamin C can be found in the brain and neuroendocrine tissues (7). Ascorbic acid is essential for the maintenance of collagen, which represents almost one-third of the body's total proteins. Collagen is a constituent protein of bones, cartilages, ligaments, cornea and eye lenses, skin, intervertebral discs, teeth, tendons, gums, blood vessels, and heart valves. Ascorbic acid is also essential for the synthesis of muscle carnitine (β-hydroxybutyric acid), which is necessary for the transport of fatty acids in mitochondria for energy production. Ascorbic acid is needed to synthesize catecholamines and ensure optimal functions of oxytocin, vasopressin, cholecystokinin, and alpha-melanotropin (8). Small amounts of ascorbic acid can prevent against the development of scurvy, and the accumulation of high levels of ascorbate in the plasma and tissues has been found to protect against oxidative damage and limit inflammation (8, 9). As previously mentioned, vitamin C concentration is higher in the brain compared to other organs; therefore, it is likely to contribute to maintaining cognitive functions (10). In newborns, vitamin C deficiency leads to memory impairments due to the decreased neurons in the hippocampus (11). In the elderly, optimum vitamin C levels can help reduce the intensity of many degenerative diseases such as Parkinson's disease, perhaps by manipulating dopamine regulation (12, 13). Vitamin C deficiency also contributes to a higher risk of stroke and associated complications in the elderly (10, 11). It has also been found that smokers are at a higher risk of developing a vitamin C deficiency and may require an additional 35 mg/day of vitamin C to be able to maintain proper vitamin C functions (13).

Another clinically important finding is that vitamin C is associated with reduced mortality in certain populations. Prospective studies found that plasma concentration of ascorbic acid was inversely related to mortality from all causes, including an array of cardiovascular diseases and ischemic heart disease (14, 15). When vitamin C was combined with vitamin E, selenium, β-carotene, and zinc, it reduced total mortality, and this mortality reduction was especially pronounced in men (16). Furthermore, prospective studies of critically ill patients showed that intravenous ascorbic acid at 3 g/day reduced multiple organ failure, ICU stay lengths as well as mortality rate (6, 17). This paper will briefly discuss the recommended amounts of vitamin C, its regulation in humans, and the role of vitamin C in oral health in an attempt to highlight the importance of vitamin C in oral health.

## Recommended Amounts, Sources, and Regulation of Vitamin C

The vitamin C requirement in a healthy adult is the amount that will compensate for metabolic losses. An average fasting vitamin C plasma level should be around 50 μmol/l (18). Daily recommendations for vitamin C are based on the individual and are different for infants, adolescents, women, men, pregnant, and lactating women (18). The Recommended Dietary Allowance (RDA) of vitamin C for adult men is 90 mg/day, and the RDA for adult women is 75 mg/day, with the upper limit being 2,000 mg/day (18).

Vitamin C is widely distributed in fresh vegetables such as broccoli, green and red pepper, tomatoes, green leafy vegetables, cauliflowers, and cabbage (1, 2, 7, 19). Fruits that are rich in vitamin C are oranges, pineapples, papaya, raspberries, lemons, strawberries, cherries, cantaloupes, grapefruits, and watermelon (1, 2, 7, 19). Potatoes have also been found to be a source of vitamin C (1, 2, 7, 19).

**Figure 1:**
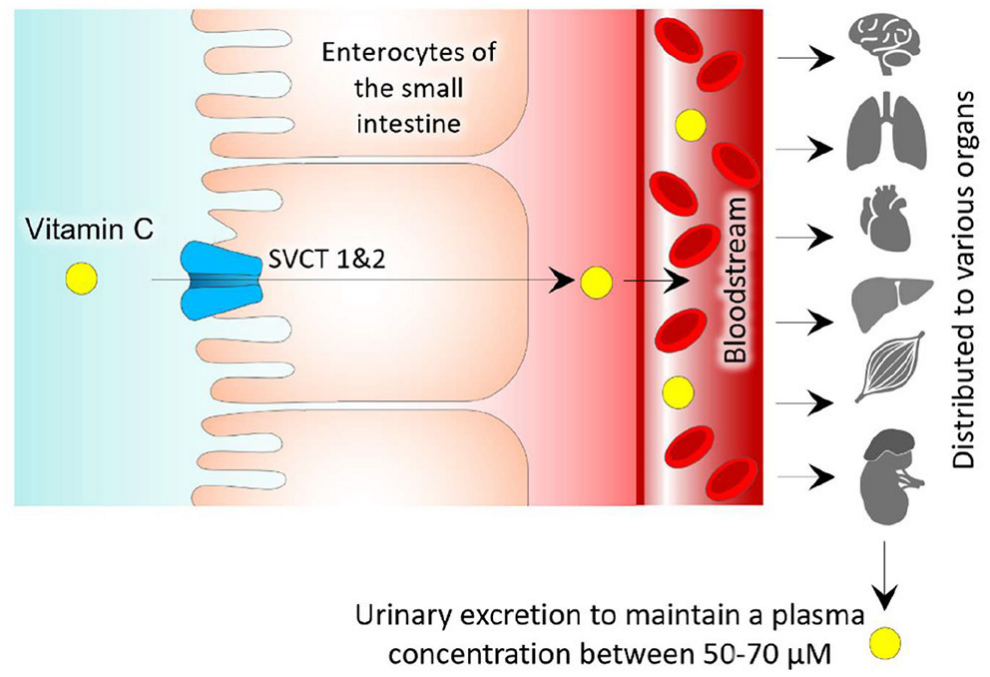
Simplified steps of regulation of vitamin C.

Under healthy conditions, the plasma concentration of vitamin C depends on dietary intake of ascorbic acid or its reversible oxidized metabolite [dehydroascorbic acid (DHAA)] (20). DHAA can be an adequate dietary source of vitamin C in humans because cellular mechanisms for transport allow DHAA to be converted into ascorbate. Additionally, DHAA has a similar bioavailability to ascorbic acid. Enterocytes absorb ascorbic acid and DHAA in the lumen of the small intestines (Figure 1). Human enterocytes have reductases that have the ability to convert DHAA into ascorbic acid. Sodium-independent carriers take up DHAA by facilitated diffusion, while ascorbic acid uses sodium-ascorbate cotransporters which are different from the ones used for DHAA. There are two isoforms of sodium-ascorbate cotransporters, SVCT1 and SVCT2 (21). SVCT1 has been shown to have a higher capacity for ascorbate transport than the other isoform due to its inherent protein structure and size (20). These transporters show relevance because SVCT1 has reduced expression in the elderly, in turn, making their daily requirements higher than younger individuals. It is also important to note that chronic inflammation of the gastric mucosa can decrease the concentration of ascorbate; therefore, patients with chronic gastritis should be potentially monitored for possible vitamin C deficiencies.

After the ascorbic acid is absorbed from the small intestines, it will enter the bloodstream and be rapidly taken up by GLUT1 transporters on erythrocytes (20–22). Ascorbic acid will then be distributed from the blood to different organs; however, the brain is a special exception in that it has a blood-brain barrier and lacks SVCT2 expression, which prevents direct transfer of ascorbic acid. Thus, ascorbic acid makes its way to the brain through the cerebrospinal fluid by going through SVCT2 receptors in the choroid plexus (21).

Because of the relatively low hydrophobicity, simple diffusion of vitamin C across the plasma membrane plays a minor role in its regulation. The transport and metabolism of vitamin C are important regulatory functions that can help concentrate intracellular levels of vitamin C and enhance its function as an enzyme cofactor and antioxidant. In humans, the plasma saturation of vitamin C is around 70 μM, and when the dose is above saturation, urinary excretion is increased, and oral bioavailability decreases, in turn permitting a sustained steady-state equilibrium (21, 22).

Adequate amounts of vitamin C in the body allow for the biosynthesis of collagen, catecholamines, and carnitine (18). Vitamin C can also help with the absorption of iron and can provide antioxidant protection. Several proteins have been identified as critical regulators of vitamin C homeostasis. For example, vitamin C transporters regulate its bioavailability in plasma and tissues. Additionally, vitamin C is used in the reduction of active redox metal factors and plays an important role in a host of endogenous stresses such as oxidative stress, infection, and inflammation (20).

## Vitamin C and Oral Health

The essential role of vitamin C in health and disease is well-studied; however, its role in oral health is not studied in similar depth and detail (23). A study done by Eydou et al. has shown that vitamin C can play a vital role in preventing the development of dental caries (24). This study revealed that there is a concentration-dependent inhibitory effect between vitamin C and *Streptococcus mutans*. S. *mutans* is a key bacterium that has been linked to the development of dental caries. As mentioned, vitamin C contributes to collagen synthesis, which is an important protein for providing tooth structure, support, and maintenance (25, 26). Vitamin C induces calcium deposition, mineralization, and reduces the risk of developing secondary caries in children (27). In a meta-analysis conducted to review dietary factors associated with dental erosion, Li et al. revealed that chewing vitamin C tablets was significantly associated with tooth wear development (28) partly due to its low pH. Despite the beneficial effect of vitamin C on dental health, oral health educators should reinforce the important oral health practices such as decreasing the time that soft drinks, fruits, and other vitamin C-containing staff remain in the mouth (28). The literature also highlights that erosive tooth wear is associated with frequent consumption of acidic fruits, juices, and chewable vitamin C with a pH lower than the normal oral pH (2 <5.5) (29).

There is scientific evidence linking periodontal disease and vitamin C deficiency (30, 31). Clinical studies have found that vitamin C depletion can cause gingival bleeding regardless of oral hygiene (32). Individuals with lower blood levels of vitamin C has presented with severe periodontal diseases compared to individuals with higher vitamin C concentration in their plasma (30). Vitamin C released from chewing gum used in healthy individuals can result in lessened supragingival calculus deposition (33). Vitamin C reduces the inflammation reaction in periodontal disease, and the administration of vitamin C supplements has been shown to improve periodontal conditions (30, 34). The periodontal healing activity is attributed to the antioxidant activity of vitamin C, and its role in collagen biosynthesis that facilitates wound healing (34). A vitamin C deficiency can result in scurvy which commonly manifests with bleeding gums and increased tooth mobility due to weakened collagen that constitutes periodontal ligament, and leads to atrophic changes of ameloblasts and odontoblasts. A clinical trial conducted by Shimabukuro and colleagues on patients with gingivitis found that spontaneous bleeding and redness of the gum could be reduced by the use of vitamin C (35). A similar reduction of gingival inflammation and bleeding following the use of vitamin C is also documented in patients with chronic gingivitis, chronic periodontitis, and type 2 diabetes (30, 36).

Vitamin C is an antioxidant that is capable of inhibiting the initiation of carcinogenesis and can help to neutralize the transformation of cells (37). Vitamin C is believed to play a protective role in patients with oral cancers. A study involving patients with oral cancers showed that patients with oral cancers had decreased saliva levels of vitamin C compared to the control group (38). A case-control study found that dietary intake of vitamin C was associated with a reduced risk of oral premalignant lesions (39). In addition, a high intake of vitamin C from natural sources (i.e., fruits, vegetables) was associated with a significantly lower risk of head and neck cancer (40). A study conducted among patients with oral cell carcinoma grade I and II revealed a marked decrease in vitamin C levels among oral cancer patients compared to the control group (37). Hence, a vitamin C deficiency is regarded as a risk factor for oral carcinogenesis. Thus, vitamin C is currently recommended as a therapeutic measure to minimize the initiation and progression of oral cancer (37, 41).

## Vitamin C Overdose

Even though the side effects of normal vitamin C intake are minor or inexistent, at large doses, calcium oxalate stones can be formed because vitamin C converts to oxalate during the elimination process. Patients with renal dysfunctions are more prone to developing calcium oxalate stones; however, it can still happen to healthy individuals at a daily dose of greater than one gram (14). The most common negative effects of high doses of vitamin C are gastrointestinal distress such as gastric pain and flatulence, nausea as well as diarrhea which appeared at the oral ingestion of a single dose of 5–10 g or daily consumption of 2 g. These symptoms usually disappear within 1–2 weeks after reducing the consumption (13, 14). Vitamin C increases iron absorption and transportation across the epithelium of the small intestines (13). This poses an additional risk to patients with sickle cell anemia, hemochromatosis, beta-thalassemia major, or sideroblastic anemia with iron overload. Additionally, in the case of a glucose-6-phosphate dehydrogenase deficiency, there is a higher risk of hemolysis (13, 14). It is advised to split the amount of ingested vitamin C into multiple doses to maintain a sustained release of the formulations in order to maintain a protective level in the plasma and reduce gastric complications. Even though vitamin C tends to be well-tolerated, it should not exceed tolerable upper intake levels, which is two grams per day for adults (13, 42).

## Summary

**Figure 2:**
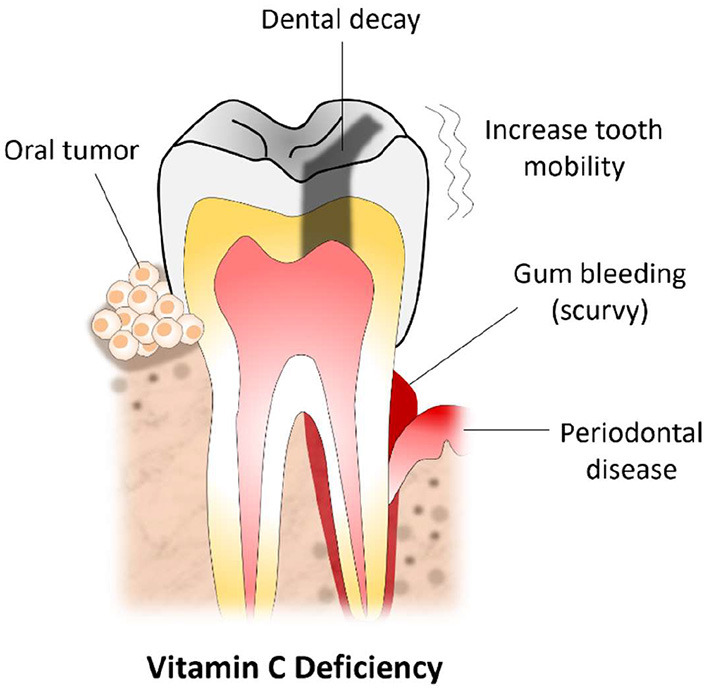
Vitamin C deficiency and commonly encountered oral lesions.

In all, this paper elaborated the recommended amounts of vitamin C, its regulation in humans, and the role of vitamin C in oral health (Figure 2). It was determined that the RDA of vitamin C for adult men is 90 mg/day, and the RDA for adult women is 75 mg/day. Vitamin C is abundant in many fresh vegetables such as broccoli, green pepper, tomatoes, and green leafy. It is also found in an array of fruits such as oranges, pineapples, papaya, and lemons.

Additionally, this paper revealed that humans could use both forms of vitamin C, ascorbate, and DHAA; however, their transport mechanisms and regulations differ slightly. It was also found that adequate amounts of vitamin C in the body are vital for the synthesis of collagen, catecholamines, and carnitine. Also, it was determined that there is a concentration-dependent inhibitory effect between vitamin C and *Streptococcus mutans*. A vitamin C deficiency can result in scurvy which can present with bleeding at the gums and increased tooth mobility. Vitamin C also plays a major role in reducing the severity of gingivitis and advanced stages of periodontal diseases. In addition, this paper highlighted that while an overdose on vitamin C is rare, it can still occur in individuals with renal dysfunctions and can potentially cause calcium oxalate stones. However, most individuals that exceed the upper limit of the RDA of vitamin C are likely to experience gastrointestinal disturbances such as gastric pain, flatulence, nausea, and diarrhea.

A future area of study includes expanding the scope of the investigation to evaluate the underlying molecular mechanism of how vitamin C reduces disease burden in chronic disorders ranging from vascular to skeletal, metabolic, neurogenerative and oral diseases.

## Author Contributions

JM, AU, and PN collected information and drafted the manuscript. JP edited the manuscript and added aditional info. MR conceptualized and reviewed the manuscript. All authors contributed to the article and approved the submitted version.

## Conflict of Interest

The authors declare that the research was conducted in the absence of any commercial or financial relationships that could be construed as a potential conflict of interest.

## Publisher's Note

All claims expressed in this article are solely those of the authors and do not necessarily represent those of their affiliated organizations, or those of the publisher, the editors and the reviewers. Any product that may be evaluated in this article, or claim that may be made by its manufacturer, is not guaranteed or endorsed by the publisher.
